# How to Design the eHMI of AVs for Urgent Warning to Other Drivers with Limited Visibility?

**DOI:** 10.3390/s23073721

**Published:** 2023-04-04

**Authors:** Dokshin Lim, Yongwhee Kwon

**Affiliations:** 1 Department of Mechanical and System Design Engineering, Hongik University, Seoul 04066, Republic of Korea; 2 Korea Product Marketing Team, Hyundai Motor Company, Seoul 06182, Republic of Korea

**Keywords:** autonomous vehicles, external human-machine interface, other road users, collision warning

## Abstract

The importance of an external interaction interface (eHMI) has grown in recent years. Most eHMI concepts focus on communicating autonomous vehicle (AV)’s yielding intention to pedestrians at a crossing. However, according to previous studies, pedestrians at a crossing rely mainly on the vehicle’s movement information (implicit communication) rather than information from eHMIs (explicit communication). This paper has the purpose of proposing a specific use case in which the eHMI of future AVs could play an indispensable role in the safety of other road users (ORUs). Often VRUs cannot see the traffic flow due to a series of parked or stopped vehicles, which is a frequent cause of fatal traffic collision accidents. Drivers may also not be able to see approaching pedestrians or other cars from the side for the same reason. In this paper, the impact of an eHMI is tested from the perspective of drivers with limited visibility when a jaywalker steps into the road. A combination of colors, shapes, and information levels is presented on an eHMI. We show that our proposed eHMI design, in the deadlock scenario of a jaywalker and a driver who both lack visibility, significantly reduced the reaction time compared to when there was no eHMI. In the experiment, the willingness to stop, varying from 0 to 5, was measured from the driver’s perspective. The results showed that most users felt uncertainty and did not move quickly when seeing the light band color alone. Textual information on the eHMI was significantly more effective in providing an urgent warning of this specific scenario than vertical and horizontal light bands with color without text. In addition, red color, blinking rapidly above 3 Hz, and egocentric messages were also necessary to reduce the PRT(perception response time). By using text-added eHMI (Vertical + Text eHMI), the mean time to achieve a score above 4 for willingness to stop was 2.113 s faster than when there was no eHMI. It was 2.571 s faster than the time until the slider of the participants reached the maximum level for willingness to stop. This is a meaningful amount of difference when considering a PRT of 2.5 s, which is the Korean road design standard. As eHMIs tend to be applied for smarter mobility, it is expected that they will be more effective in preventing accidents if the eHMI is standardized in autonomous driving level 2 to 3 vehicles driven by humans before fully autonomous driving becomes a reality.

## 1. Introduction

### 1.1. Definition of Terms

#### 1.1.1. Automated Vehicle vs. Autonomous Vehicle

Autonomous vehicles (AVs) are vehicles capable of sensing their environment and moving safely without human input. They are also known as connected and autonomous vehicles (CAVs), driverless vehicles, robotic vehicles, or vehicles that exhibit SAE Level 5 automation [1]. Automated vehicle is a wider term meaning any vehicle equipped with driving automation technology for longitudinal and lateral vehicle control, which can free the driver from the driving task—at least in some driving situations, as defined in SAE J3016 [2,3,4]. The former acronym ‘AV’ (autonomous vehicle) is used in this paper because our research focuses on the ability to interact with all the elements that make up the ecosystem, including road users [5]. AVs can detect and classify objects in the vicinity and can notify the driver and other road users about the situation with respect to, for example, pedestrian detection state (pedestrians with intention to cross, pedestrians that stop suddenly or start running), detection of a traffic signal, and detection of objects on the road, among others. AVs can take real-time control of certain operations with the aim of avoiding accidents [6].

#### 1.1.2. iHMI vs. eHMI

With the advent of AVs, the importance of an external interaction interface (eHMI) has grown in recent years. The generally used term HMI (human-machine interface) is divided into iHMI (internal human-machine interface) and eHMI due to vigorous development of eHMIs in academic research as well as in industry applications. An iHMI is an internal human-machine interface positioned in the vehicle’s interior to communicate with the on-board user (or driver, when the driving is not automated); an eHMI is an external human-machine interface positioned on the vehicle’s exterior to communicate with surrounding traffic participants [7]. More specifically, an eHMI is defined as ‘any interface perceivable from the exterior of a vehicle to communicate and/or interact with another human road user, including conventional methods, such as honks or the turn indicator, but also novel concepts projecting images on the ground’, or simply as an ‘external human-machine interface positioned on the vehicle’s exterior to communicate with surrounding traffic participants’ [8,9,10].

If a driver is not present or inattentive, the AV has no means to communicate explicitly with other road users (ORU), other than using available exterior lights, the use of which is defined in traffic regulations. Although eHMIs have the potential to benefit road safety in the current human–driver paradigm, they will become critical with the emergence of AVs when current channels of communication between drivers and road users, such as eye-contact and gestures, are no longer feasible.

#### 1.1.3. VRU vs. ORU

Vulnerable road users (VRU) are, in general, defined as ‘non-motorized road users, such as pedestrians and cyclists, as well as motorcyclists and persons with disabilities or reduced mobility and orientation’ [11]. According to WHO’s Global Road Safety Report [12], more than 50% of all road traffic deaths occur among motorcyclists (23%), pedestrians (22%), and cyclists (5%). A road traffic crash is defined as a collision or incident that may or may not lead to injury, occurring on a public road, and involving at least one moving vehicle [13]. For the purposes of improving safety of road users from road traffic crashes, a vulnerable road user can be more specifically defined as a road user who is present in a crash involving vehicles which do not have a protective shell [14].The relationship between the pedestrian and the vehicle involved in a road traffic crash, including the active or passive perception of the other party, and the ability to see and be seen on the part of both pedestrians and vehicles, is becoming a key factor in the prevention of accidents. The pedestrian must be seen by the driver and the vehicle by the pedestrian. This paper demonstrates how an AV plays the role of bridging the gap when the driver and the pedestrian lack sight of each other.

Despite the prominence of vulnerable road users, many of whom cannot afford or do not have access to the safest vehicles equipped with active safety features to avoid collisions, such as automated braking. The coming wave of AVs is expected to be used to protect VRUs more [3,15]. It depends largely on how AVs communicate and interact with other road users.

When living with AVs in the future, the concept of VRUs needs to be extended to all other possible road users, including drivers of vehicles operated manually.

### 1.2. Related Work

#### 1.2.1. Taxonomy and State-of-the-Art Reviews

A variety of novel concepts have been introduced, as shown in extensive analyses of eHMI taxonomies [15,16,17,18,19]. The recent advent of eHMI concept proposals has seen a recurrence in certain patterns of design choices for communication of right-of-way negotiation. Dey et al. [16] proposed an 18-item taxonomy by clustering 70 eHMI concepts and identified the following 12 design patterns:

1. Projections on the road;
2. Symbols—commonly understood traffic symbols;
3. Text—message script in characters or numbers;
4. Smile—anthropomorphic smile element to indicate friendly (yielding) behavior;
5. Eyes—anthropomorphic eyes to show the AV’s situational awareness;
6. Other anthropomorphic designs—‘gestures’, avatars, or other elements that are approximations of human communication behavior;
7. Abstract lighting element: one-dimensional light bar or segment;
8. Abstract lighting element: two-dimensional display;
9. Abstract lighting element: tracker—to show the situational awareness of the car in its environment;
10. Audio;
11. Infrastructure elements;
12. Mobile and/or wearable devices.

In terms of the modality of communication, our research primarily focuses on the visual (representing 97% of existing concepts). Existing eHMIs have mostly used abstract information through lights and displays (69%), followed by symbols (29%), text (23%), and anthropomorphic features (14%) [16]. Lim and Kim [20,21] proposed a reference eHMI combining a one-dimensional light status bar and a two-dimensional area that presents textual or graphical information. They incorporated eight of the 12 design patterns above (excluding 1. Projections on the road, 6. Other anthropomorphic designs, 11. Infrastructure elements, and 12. Mobile and/or wearable devices), and proposed a scalable UI system to meet various contexts of use. This paper also proposed a composite of visual representations—both abstract and textual information on a UI system.

Regarding the message using existing eHMI concepts, communicating AV’s yielding intention to pedestrians at a crossing is the most common scenario. Interactions with road users other than pedestrians are rather unexplored. According to Dey et al. [16], among 70 concepts analyzed, pedestrians were addressed by 64 concepts (91%), while interactions with manually operated vehicles were only addressed by 10 concepts (14%).

This paper has the purpose of considering an unexplored scenario and proposes a specific use case in which eHMIs for future AVs play an indispensable role in the safety of other road users (ORUs). The future AV serves as a medium for the communication between pedestrian and driver in a manually operated vehicle who both lack visibility.

#### 1.2.2. Usefulness of eHMIs

A number of studies have shown that pedestrians at a crossing rely primarily on a vehicle’s movement information rather than information from novel eHMIs. The majority of them, however, report that communicating the intent of the AV using an external interface can contribute to a positive interaction experience [9,22,23,24,25,26,27]. Other studies have concluded that the car’s motion may suffice [28,29] or leave the judgment open [30]. The majority of experts in the field of AVs suggest that eHMIs will enhance future AV–ORU interaction, but note that implicit communication will remain dominant, advising against text-based and instructive eHMIs [18,19]. Learned positive experiences are important for building trust and acceptance toward new technologies [31]. That is why it is critical to use the surprise factor when using eHMIs when AVs are first introduced to the market. If the pedestrian crossing scenario is a ‘nice to have’, more useful user scenarios should arise.

Around the world, autonomous cars could save 10 million lives per decade, creating one of the most important public health advances of the 21st century [32]. From the perspective of public health, the overarching goal is to transform the current approach to automotive safety from reducing injuries after collisions to complete collision prevention [33]. eHMIs could be used for improving the active safety not only of passengers, but also other road users based on AV sensors and technology. For this reason, our study deals with a specific use case in which the eHMI of future AVs could play a more important role in collision prevention.

Often road users, whether pedestrians or drivers, lack sight of the traffic flow due to a series of cars in line (e.g., parked, stopped, and driving vehicles), which is a frequent cause of pedestrian-vehicle collision accidents. In a report that analyzed pedestrian accidents in Korea, it was found that a limited view of drivers due to a line of vehicles was associated with 23.9% of analyzed accidents [34]. Looking further at this type of accident, sport utility vehicles (SUVs) and vans were associated with 41.5% of the pedestrian accidents due to the limited view of drivers because such vehicles can obscure the view of other drivers as well as pedestrians more than low-height vehicles. The height of AVs tends to be more elevated than that of normal passenger cars due to sensors mounted on the roof and a battery platform placed on the bottom. This raises concerns about a higher risk that other drivers and pedestrians around future AVs will be subject to more limited visibility of road traffic. The role of eHMIs will become more critical than ever in this situation.

In this paper, the impact of an eHMI was tested from the perspective of drivers with limited visibility when a jaywalker steps into the road. A combination of colors, shapes, and information levels was presented on an eHMI. We showed that our proposed eHMI design in the deadlock scenario between a jaywalker and a driver who both lack visibility, significantly reduced the reaction time compared to when there was no eHMI. The willingness-to-stop data (to infer the PRT, defined later) from the perspective of drivers of manually driven cars were logged using a coded slider, while the test participants watched the video in the laboratory. Our goal was to present an eHMI design that could maximize the effect of preventing collision accidents in environments where it is difficult for drivers to predict the appearance of a pedestrian due to limited visibility.

## 2. Assumptions

### 2.1. Target Road Users

The main type of road user addressed in the study is drivers of manually operated vehicles. According to Dey et al. [16], eHMIs address pedestrians in 91% of cases, while cyclists are targeted by only 23%, and manually operated vehicles by 14%, which seems much less than is needed given the high level of influence of other drivers who share public roads with AVs [20]. Moreover, 60% targeted pedestrians only, while 31% targeted pedestrians and at least one other type of road user. There exists much room for eHMI design to contribute to the safety of other drivers of manually driven cars on the road.

**Figure 1:**
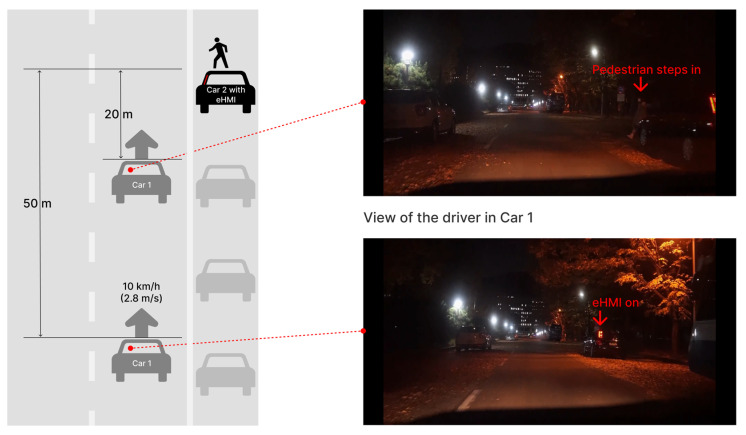
Test scenario filmed at night.

This study proposes an eHMI that communicates mainly with other drivers in manually operated vehicles (Car 1), nearby AVs (Car 2 + eHMI), as well as pedestrians who are exposed to potential risk of collision due to obscured visibility, as shown in Figure 1.

### 2.2. eHMI Design

**Figure 2:**
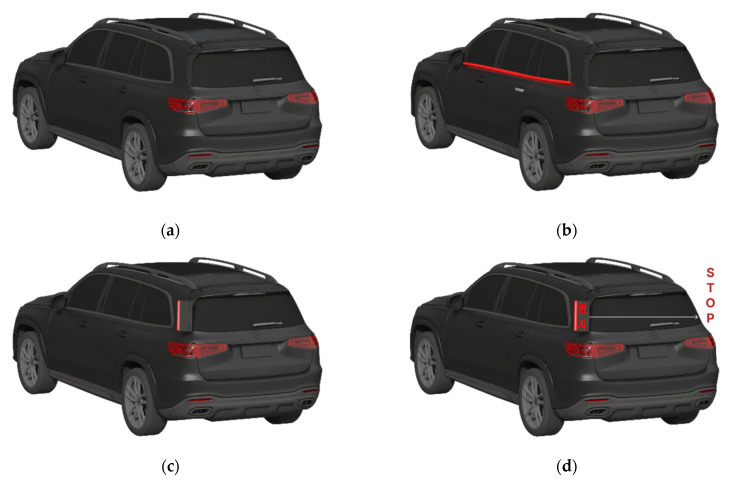
Four tested cases (digital prototype): (**a**) No eHMI; (**b**) Horizontal light band; (**c**) Vertical light band; (**d**) Vertical light band plus text “STOP” written in Korean.

Our eHMI concept was demonstrated on a typical passenger car type (not shuttle buses or heavy cars) and the HMI placement was on the vehicle (not a projection on the road, on the infrastructure, or on pedestrians), as shown in Figure 2. Naturally, auditory information from the eHMI was not considered in this case for other drivers, while the multi-modal aspect of the eHMI was highly important from the perspective of the pedestrian, as shown in Figure 1.

Considering the test scenario in Figure 1, the side and corner within the greenhouse area of the vehicle were considered for placement of the eHMI. Its vertical position was located high to meet the eye level of other drivers in the car. The primary modality of communication with drivers in the other car was naturally visual because the sound emitted from eHMIs mounted on the AV was not certain to reach other drivers who stayed within the interior of the car. Abstract or text type visual information were considered, while keeping the possibility of using both open. The egocentric message “STOP” [35] is written in red color [36] (see in Figure 2d) and the light band blinked at 3 Hz [37].

### 2.3. Evaluation of the Concept

Evening and nighttime conditions were assumed to maximize the risk of obscured vision of the driver and of the pedestrian. For the sake of safety, the scenario was simulated outdoors with a physical prototype and recorded in a video file. Later the video was shown to the test participants on the computer monitor indoors.

## 3. Methods

### 3.1. Apparatus

**Figure 3:**
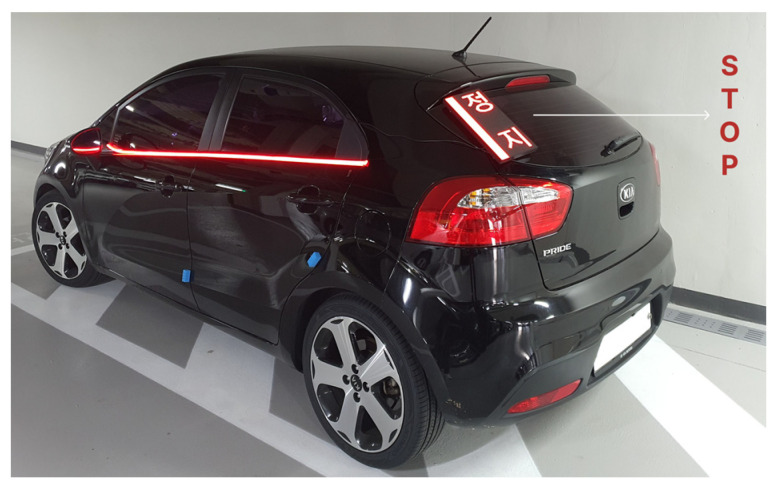
eHMI implemented on a real car used for recording (physical prototype).

An LED panel that fitted the height of the rear glass was mounted in the corner. The display size of the LED panel was 164 mm (width) × 320 mm (height). A 2.5 pitch LED with a resolution of 64 × 128 pixel was used. A total of 12 pixels were used as a red light blinking, while the rest were used to display text. A light strip was installed along the bottom line of the greenhouse area on the side. Figure 3 shows the physical prototype to demonstrate how it is seen on a passenger car type vehicle.

**Figure 4:**
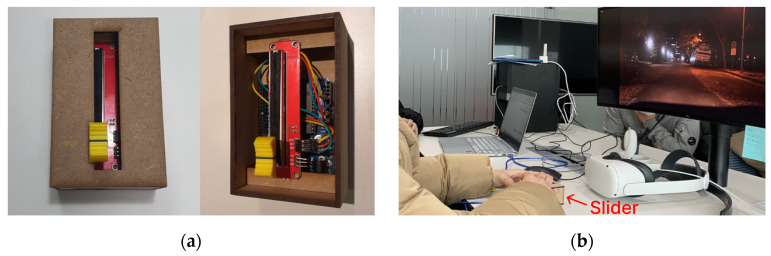
Test settings: (**a**) The input device—a continuous slider used to receive driver feedback to a sudden pedestrian stepping in; (**b**) Participant using the slider while watching the video in the lab.

A “feeling-of-safety” slider [38] was made using Arduino Uno and coolterm ver 1.9.0 (see Figure 4a). The purpose of using this slider was to record the data on how participants immediately reacted while watching the video on the monitor. As Walker et al. demonstrated in their work, we used a slider type input method as a way of evaluating the interaction between AVs and road users in a quantifiable, continuous data and standardized way. The slight difference corresponds to the reverse way of thinking—the willingness to stop versus the feeling of safety.

The arrangement and movement of the vehicles are shown in detail in Figure 1. It was important that all participants experienced the exact same view at the same speed of driving. Thus, it was preferable to use a filmed movie rather than testing in a real situation in which we could not avoid inconsistency. The filmed video was shown on a 34-inch-wide monitor with 21:9 ratio, as shown in Figure 4b. The length of the video was 25 s. Adjustable resistance data was gathered every 10 millisecond(s) on a scale of 0.01 from 0 to 5.

### 3.2. Procedure

The tests were within-subject. Four videos from the view of the driver sitting in Car 1 containing different cases of Car 2 (refer to Figure 1) were shown to each participant. Participants watched the videos completely from the beginning to the end and moved the slider as their level of willingness to stop changed. The order of the four videos shown was randomized. All participants had enough time each trial to use the slider and adjust the movement of the slider, starting from 0 to the maximum level 5, according to how they felt so that they could get used to it. When participants finished watching all four videos, they were asked questions about how they perceived the message and felt about the situation in the videos in a semi-structured interview.

### 3.3. Data and Analysis

Driver perception response time (PRT) was defined as the time that it takes a driver to perceive and respond to an unexpected situation, which is of considerable interest to those who design roadway systems, as well as in many instances of litigation resulting from motor vehicle crashes [39]. It is commonly known as the reaction time and can be defined as the time that elapses from the instant that a driver recognizes the existence of a hazard in the road to the instant that the driver takes appropriate action, for instance, by applying the brakes. In this study, the PRT was inferred from the continuous measurement of participants’ willingness to stop logged from the slider. According to the Korean road design standard, the recommended PRT applicable to 95% range drivers is considered to be 2.5 s, when the speed of driving is at the level of 30 km/h [40,41]. The same PRT of 2.5 s applies in many countries, such as USA, Canada, Australia, and South Africa [42], while a shorter time—2.0 s—applies in European countries.

The data from the slider varied from 0 to 5 during the time that each video played from 0 to 25 s. We inferred the PRT from the time that it took from the moment when the eHMI was activated to the moment when the participant moved the slider over point 4 of their willingness to stop. This was based on the assumption that level 4 (within the scale of 0—5) was high enough for their willingness to stop. Another analysis used the time that it took from the moment when the eHMI was activated to the moment when the participant moved the slider to the maximum level within their personal range (even though the maximum level for her or him was lower than 4), which allowed each participant to subjectively gauge their willingness to stop. We analyzed the time until the willingness to stop logged from when the slider reached a certain level, such as level 4, or each one’s maximum (dependent variable) according to four tested cases (independent variables).

The statistical analysis and most of the graphical representations were performed using Jamovi version 1.6 [43]. Analysis of variance was used to compare the estimated marginal means [44] for the four tested cases as well as for sub-groups of participants (e.g., by age, gender, and frequency of driving).

### 3.4. Participants

A total of 31 people (16 females and 15 males) ranging in age from 20 to 31 years with a mean age of 24.84 years (SD: 2.2375, Median: 25) participated in the test in November 2021. The participants were recruited via university emails and personal invitations. Twenty-six (83.9%) had a driver’s license and the other five persons (16.1%) did not have a license. The participants’ driving frequency was investigated in the pre-questionnaire, and was divided into three levels (see Table 1).

## 4. Results

### 4.1. Willingness to Stop

**Figure 5:**
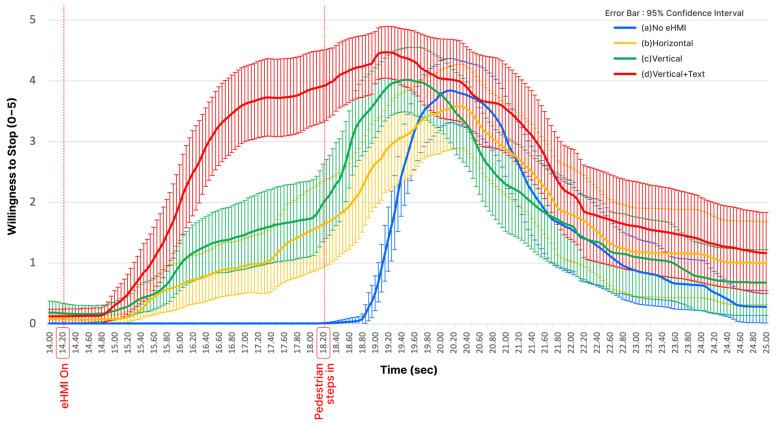
Willingness to stop (continuous data logs from 0 to 5) by four tested cases.

Figure 5 illustrates the data logged from the slider. The x-axis is the continuous rating of the participants’ willingness to stop. The y-axis is the time elapsed after the movie played. The legend is coded in different colors according to eHMI design: (a) No eHMI in blue, (b) Horizontal in yellow, (c) Vertical in green, and (d) Vertical + Text (vertical light band plus text case) in red.

Two milestones are marked on the x-axis. The first one “eHMI On” is the time when the eHMI was activated (14.20 s in the video play time). The second one “Pedestrian steps in” was the time when the pedestrian was actually visible on the video screen (18.20 s in the video play time).

It was observed that in the case of (a) No eHMI, the reaction of test participants occurred only after the pedestrian was visible. As expected, in three cases with eHMIs, the reaction of the participants was faster than the case (a) No eHMI. Judging from the data, (d) Vertical + Text was the most effective way to make other drivers pay attention faster when visibility was obscured. Interestingly, two cases ((b) Horizontal and (c) Vertical), in which the eHMI was abstract, the willingness to stop occurred at significantly slower speeds than when there was “STOP” text written (see the steep slopes in red and blue compared to the gentle slopes in yellow and green).

To infer the driver’s perception response time (PRT) from the data above, we compared how quickly the participants reached a sufficiently high willingness to stop (4 in our analysis). The other way used was to compare the time it took for participants to reach the maximum level after the test began. Using both approaches, similar results were derived.

#### 4.1.1. Time until over 4 of Willingness to Stop Reached

**Figure 6:**
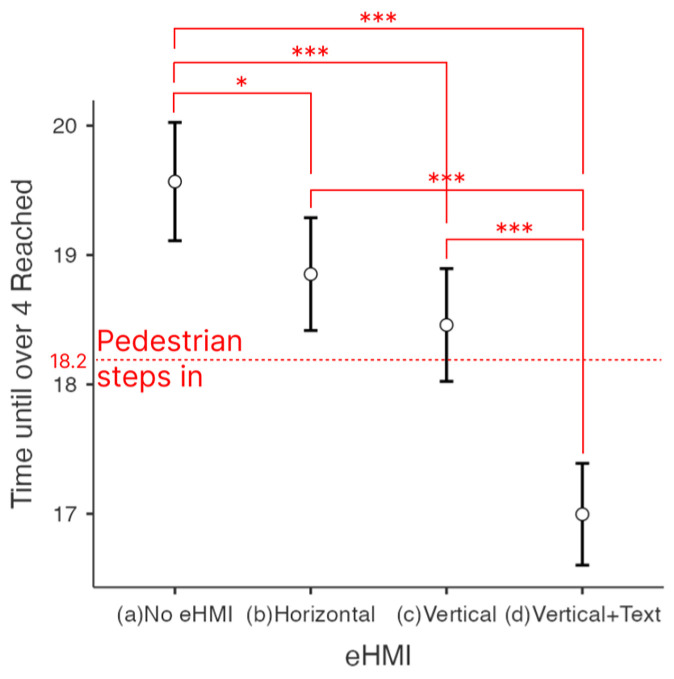
Response time (s): Time until over 4 reached. * *p* < 0.05, *** *p* < 0.001.

As explained earlier, the first way to analyze the data was to compare the time it took for participants to reach 4 for the first time after the test began. Variance might exist depending on individuals. For example, there were participants whose maximum level did not go higher than 4. There were only 20 participants who raised the slider over 4 for (a) No eHMI, 22 for (b) Horizontal, 22 for (c) Vertical, and 27 for (d) Vertical + Text. In other words, only 64.5% of the participants raised the slider over 4 when there was no eHMI, compared to 78.9%, on average, when there was eHMI. In the first analysis in Figure 6, in the case of (a) No eHMI, it took, on average, 19.6 s to reach level 4 of the willingness to stop (*n* = 20), 18.9 s (*n* = 22) for (b) Horizontal, 18.5 s (*n* = 22) for (c) Vertical, and 17.0 s (*n* = 27) for (d) Vertical + Text.

The following analysis of the time to reach 4 of the willingness to stop scale excludes the data of participants whose highest scores were lower than 4. The comparisons are based on the estimated marginal means in Table 2.

The post hoc comparison is shown in Table 3. In all cases (b), (c), and (d), the time until over 4 of the willingness to stop scale was reached was faster than case (a), which means that the existence of eHMI in any form was effective. The differences between (d) Vertical + Text and all others ((a) No eHMI, (b) Horizontal and (c) Vertical) were all significant (*p* < 0.05). This means that the willingness to stop point over 4 was reached significantly faster for the case of (d) Vertical + Text.

#### 4.1.2. Time until the Maximum Level of the Willingness to Stop Scale Was Reached

**Figure 7:**
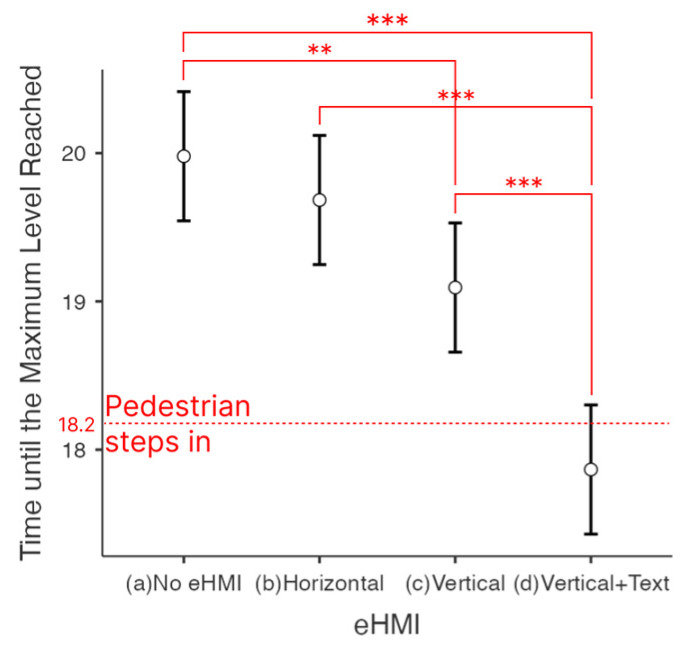
Response time (s): Time until the maximum level was reached. ** *p* < 0.01, *** *p* < 0.001.

In the second analysis in Figure 7, the mean time to reach each maximum level was compared. The marginal means were 20.0 s for (a) No eHMI, 19.7 s for (b) Horizontal, 19.1 s for (c) Vertical, and 17.9 s for (d) Vertical + Text. In this case, data from all participants (*n* = 31) were considered (see also Table 4).

The only difference was found in the variance of the two cases (a) No eHMI and (b) Horizontal. Comparing the times until the participants reached the maximum level of willingness to stop, there was no significant difference between the two cases. In the first analysis (Section 4.1.1), data staying under 4 all the way were excluded. Thus, it seems that the first means of analysis resulted in clearer differences than the other approach (Section 4.1.2) of counting all data, even though the internal variances were small.

In both Table 3 and Table 5, between the two tested cases (b) Horizontal and (c) Vertical, there was no significant difference. Thus, we cannot expect that using this type of abstract eHMI people will universally pay attention to a certain level and initiate braking action quickly enough.

### 4.2. Effect of the eHMI

The previous analysis confirmed that there was a positive effect of eHMIs compared to the no eHMI case. The effect size depended on each eHMI design varied, however. It is clear from Figure 5, Figure 6 and Figure 7 that the willingness to stop occurred faster for (b) Horizontal, (c) Vertical, and (d) Vertical + Text than the (a) No eHMI case. However, in the second analysis, when using the time until the maximum level of the willingness to stop was reached, (b) Horizontal was not significantly faster than the (a) No eHMI case.

### 4.3. Effect of the Text

Figure 6 and Figure 7 clearly show that only the mean of the case (d) Vertical + Text was less than 18.2 s (the moment when the pedestrian steps in the movie). This means that although the time was faster when horizontal and vertical light bands were presented than when there was no eHMI, the speed of feeling the need to stop was not enough to prevent collision. The comparison of (c) Vertical to (d) Vertical + Text, and (b) Horizontal to (d) Vertical + Text, were significant (*p*-value < 0.001) in both analyses, which means that the presence of textual information resulted in faster reaction time. The mean difference was 2.571 s (refer to Table 3) when Vertical + Text was compared to No eHMI. Analyzing the time until the maximum was reached individually produced similar results; the difference was 2.113 s (refer to Table 5) when comparing Vertical + Text to No eHMI.

### 4.4. Effect of the Frequency of Driving

**Figure 8:**
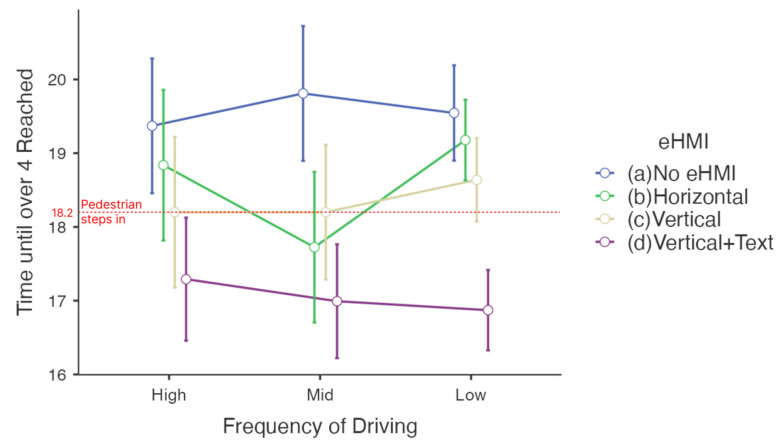
Time until level 4 of the willingness to stop scale was reached by frequency of driving (s).

There was no cross-effect observed of eHMI by frequency of driving (*p* = 0.337 in Table 6). Different behavior according to frequency of driving was somewhat evident though, as shown in Figure 8.

The quantity of data was not sufficient to represent each group according to frequency of driving. More than half of the participants belonged to the low frequency group: 18 participants out of 31 (58.06%). It was notable that this low frequency group showed significantly faster reaction times when text information was given.

Within the low frequency of driving group (*n* = 18), the differences between Vertical + Text and the other three cases were all significant (see Table 7). It can be said that abstract information on the eHMI was even more ambiguous to people who did not drive frequently or did not have a license to drive and it was better for them have clear text written on the eHMI. The mean differences between the three eHMIs (No eHMI, Horizontal and Vertical) without text were longer than for the eHMI with text (Vertical + Text) by 2.674, 2.307 and 1.767 s, respectively.

## 5. Discussion

Semi-structured 1:1 interviews with the participants revealed that some of them did not even notice the existence of the light band (Horizontal and Vertical eHMIs) although it blinked at 3 Hz. To deliver urgent messages to the drivers, blinking at 3 Hz was used in Kwon’s experiment [21], or even more frequently, to better draw attention.

For some participants who noticed the eHMIs, the horizontal light band was perceived as an added direction indicator or a decoration in tune with the exterior of the vehicle. Between the horizontal and vertical light bands we used, the vertical one seemed to be better qualified as an eHMI to emit new information to road users in our test scenario (refer to Figure 1). There was a subjective view though that the vertical band looked like a bat used by the police, which is not bad for the purpose of warning.

PRT consists of four serial steps that include sensory detection, perception (information processing), decision making, and, finally, control response [46]. It is aligned with the well-known Wickens’ four stages of human information processing [47]. In general, symbols and light colors are expected to be perceived quickly. Red color universally draws the attention of people quickly. However, abstract and graphical information is not always evident. Icons can be recognized quickly at a glance (if well designed), which is particularly true for standard icons that people have seen and used before. There is no need to translate icons for international users, provided that the icons are mindful of cultural differences. Icons are not always understood if they are not labelled [48]. A user’s understanding of an icon is based on previous experience. Due to the absence of a standard usage for most icons, text labels are necessary to communicate the meaning and reduce ambiguity [49]. This is the case for eHMI design for AVs, too [50]. In some familiar and representative traffic use cases, there are well known universal symbols (e.g., green man vs. WALK at a crossing [15,51,52]). In contrast, there are many traffic situations that drivers encounter but for which there are no well-known graphic symbols, as in the specific case that this paper dealt with. General symbols, such as a diagonal line to mean “Forbidden”, are not always applicable in diverse traffic situations. In some specific use cases, text information effectively provides clarity of information. However, those designing explicit signals should focus on identifying very specific instances that require additional signaling, rather than assuming explicit textual information will be necessary in all situations.

**Figure 9:**
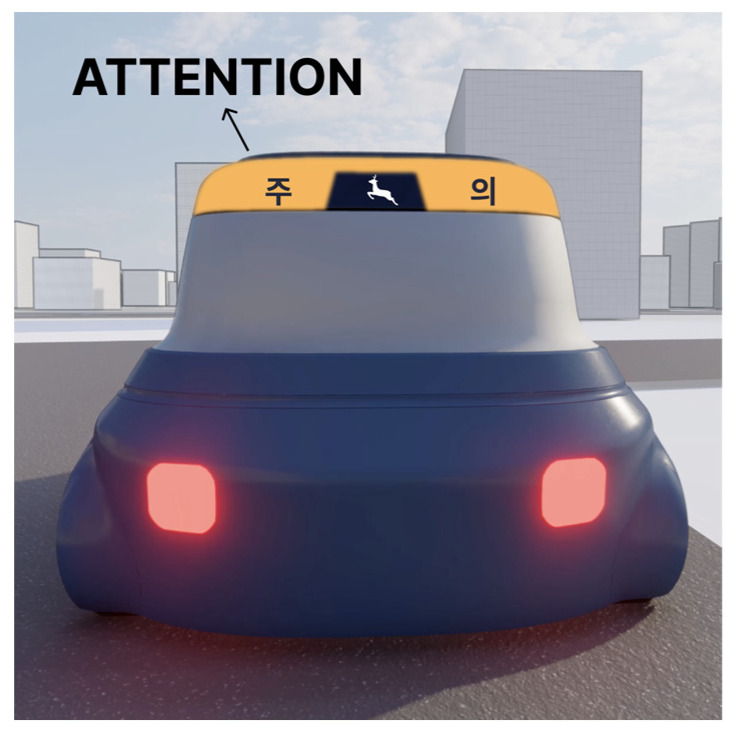
“Deer crossing” sign symbol plus text “ATTENTION” written in Korean.

Using familiar road sign symbols on the eHMI of AVs may create confusion [20]. For example, the same deer crossing symbol in Figure 9 may have different meanings. When using it on an eHMI, as in Figure 9, it will be 100% certain (unless there is an error in the AV’s detection) that there will be a deer crossing ahead. Thus, drivers following in the vicinity of the AV need to brake immediately. The familiar deer crossing sign [53,54] is posted in areas that are known to have deer crossing the road. When approaching a deer crossing sign, the driver should be alert and prepared to stop (not having to stop immediately) for any wildlife that may decide to cross the road ahead of them. For this reason, using familiar road sign symbols needs to be used with caution when applied on the eHMI. In this paper, the eHMI design alternatives in Figure 2 do not consider the familiar “pedestrian crossing” road sign symbol to avoid this confusion and to deal with the specific context in which the visible space is very limited due to a series of parked cars on the side near the curb.

## 6. Conclusions

One of the common types of visual interfaces are light bands that communicate with lighting colors, and displays that present textual or graphical information. Most eHMI concepts focus on communicating autonomous vehicle (AV)’s yielding intention to pedestrians at a crossing. However, according to previous studies, pedestrians at a crossing rely mainly on the vehicle’s movement information (implicit communication) rather than information from eHMIs (explicit communication). Unlike previous work, this paper considered a hybrid situation where a manually operated car, a jaywalker, and a parked autonomous vehicle interact. In this specific use case, the eHMI design for future AVs will play a critical role in improving the safety of pedestrians. Often vulnerable road users (VRUs) lack sight of the traffic flow due to a series of parked or stopped vehicles, which is a frequent cause of fatal traffic collision accidents. Drivers can also not have sight of approaching pedestrians or cars approaching from the side for the same reason. The tested eHMI significantly reduced the perception response time (PRT) of drivers of manually driven cars as inferred from testing in the laboratory. Though limited to in-laboratory testing, the results have implications for researchers exploring the roles that future AVs can play in urban environments.

A combination of colors, shapes, and information levels was presented on an eHMI. Through this, we sought to present an eHMI design that could maximize the effect of preventing collision accidents in environments where it is difficult for drivers to predict the appearance of a pedestrian due to limited visibility. Ultimately, we expect that the learned positive experiences in such a high-risk use case will help road users to build trust and acceptance toward AVs.

In the experiments undertaken, the willingness to stop, varying from 0 to 5, was measured from the driver’s perspective. The results showed that most users felt ambiguity and did not move rapidly in response when seeing the light band color alone. Textual information presented on the eHMI was more effective in providing an urgent warning of this specific scenario than vertical and horizontal light bands with color without text. In addition, red color, blinking rapidly above 3 Hz, and personally directed messages were also necessary to reduce the PRT. When all these factors were present, the PRT was reduced from 2.11 s to 2.57 s. This corresponds to the PRT of 2.5 s, which is the Korean road design standard. Applying these guidelines, providing warnings to other drivers via an eHMI is an effective solution.

As eHMIs tend to be applied for smarter mobility, it is expected that they will be more effective in preventing accidents if the eHMI is standardized in autonomous driving level 2 to 3 vehicles driven by humans before fully autonomous driving becomes a reality.

## Figures and Tables

**Table 1:** Participants’ frequency of driving (*n* = 31).

Categories	Frequency of Driving	Frequency	Percentages
High	More than 5 times a week	2	23.58%
	3~4 times a week	1	
	1~2 times a week	4	
Mid	2~3 times a month	5	19.35%
	less than once a month	1	
Low	Have a license to drive but rarely drive	13	58.06%
	Have no license to drive	5	

**Table 2:** Estimated marginal means of time until over 4 reached of the willingness to stop scale by tested cases (seconds).

Tested Cases	Mean	SE	95% Confidence Interval	
			Lower	Upper
(a) No eHMI	19.6	0.230	19.1	20.0
(b) Horizontal	18.9	0.219	18.4	19.3
(c) Vertical	18.5	0.219	18.0	18.9
(d) Vertical + text	17.0	0.198	16.6	17.4

**Table 3:** Post hoc comparison of the time until over 4 reached of the willingness to stop scale.

Comparison		Mean Difference	SE	df	t	*p*
eHMI	eHMI					
(a) No eHMI	(b) Horizontal	0.715	0.318	87.0	2.251	0.027 *
	(c) Vertical	1.108	0.318	87.0	3.489	<0.001 ***
	(d) Vertical + text	2.571	0.303	87.0	8.475	<0.001 ***
(b) Horizontal	(c) Vertical	0.393	0.310	87.0	1.268	0.208
	(d) Vertical + text	1.856	0.295	87.0	6.284	<0.001 ***
(c) Vertical	(d) Vertical + text	1.463	0.295	87.0	4.953	<0.001 ***

Note. Comparisons are based on estimated marginal means. * *p* < 0.05, *** *p* < 0.001.

**Table 4:** Estimated marginal means of time until the maximum level of the willingness to stop was reached by tested cases (seconds).

Tested Cases	Mean	SE	95% Confidence Interval	
			Lower	Upper
(a) No eHMI	20.0	0.220	19.5	20.4
(b) Horizontal	19.7	0.220	19.2	20.1
(c) Vertical	19.1	0.220	18.7	19.5
(d) Vertical + text	17.9	0.220	17.4	18.3

**Table 5:** Post hoc comparison of the time until the maximum of the willingness to stop scale was reached.

Comparison		Mean Difference	SE	df	t	*p*
eHMI	eHMI					
(a) No eHMI	(b) Horizontal	0.295	0.311	120	0.949	0.345
	(c) Vertical	0.885	0.311	120	2.846	0.005 **
	(d) Vertical + text	2.113	0.311	120	6.790	<0.001 ***
(b) Horizontal	(c) Vertical	0.590	0.311	120	1.897	0.060
	(d) Vertical + text	1.818	0.311	120	5.841	<0.001 ***
(c) Vertical	(d) Vertical + text	1.227	0.311	120	3.944	<0.001 ***

Note. Comparisons are based on estimated marginal means. ** *p* < 0.01, *** *p* < 0.001.

**Table 6:** ANOVA—the time until over 4 of the willingness to stop scale was reached [45].

Factors	Sum of Squares	df	Mean Square	F	*p*
eHMI	67.309	3	22.436	21.310	<0.001
Frequency of Driving	2.047	2	1.023	0.972	0.383
eHMI × Frequency of Driving	7.324	6	1.221	1.159	0.337
Residuals	83.174	79	1.053		

**Table 7:** Post-hoc comparison of the time till reach the maximum of the willingness to stop within the low frequency of driving group.

Comparison		Mean Difference	SE	df	t	*p*
eHMI	eHMI					
(a) No eHMI	(b) Horizontal	0.366	0.425	79.0	0.863	0.999
	(c) Vertical	0.907	0.432	79.0	2.100	0.624
	(d) Vertical + text	2.674	0.425	79.0	6.293	<0.001 ***
(b) Horizontal	(c) Vertical	0.540	0.395	79.0	1.367	0.966
	(d) Vertical + text	2.307	0.388	79.0	5.949	<0.001 ***
(c) Vertical	(d) Vertical + text	1.767	0.395	79.0	4.471	0.001 **

Note. Comparisons are based on estimated marginal means. ** *p* < 0.01, *** *p* < 0.001.

## Data Availability

The data is available upon request from the authors.
